# NiO Nanosheets Coupled With CdS Nanorods as 2D/1D Heterojunction for Improved Photocatalytic Hydrogen Evolution

**DOI:** 10.3389/fchem.2021.655583

**Published:** 2021-04-15

**Authors:** Lin Wei, Deqian Zeng, Zongzhuo Xie, Qingru Zeng, Hongfei Zheng, Toyohisa Fujita, Yuezhou Wei

**Affiliations:** ^1^Guangxi Key Laboratory of Processing for Non-ferrous Metals and Featured Materials, School of Resources, Environment and Materials, Guangxi University, Nanning, China; ^2^Collaborative Innovation Center of Chemistry for Energy Materials, College of Materials, Xiamen University, Xiamen, China

**Keywords:** NiO nanosheets, CdS nanorods, nanocomposites, p-n heterojunction, photocatalytic hydrogen generation

## Abstract

Designing low-cost, environment friendly, and highly active photocatalysts for water splitting is a promising path toward relieving energy issues. Herein, one-dimensional (1D) cadmium sulfide (CdS) nanorods are uniformly anchored onto two-dimensional (2D) NiO nanosheets to achieve enhanced photocatalytic hydrogen evolution. The optimized 2D/1D NiO/CdS photocatalyst exhibits a remarkable boosted hydrogen generation rate of 1,300 μmol h^−1^ g^−1^ under visible light, which is more than eight times higher than that of CdS nanorods. Moreover, the resultant 5% NiO/CdS composite displays excellent stability over four cycles for photocatalytic hydrogen production. The significantly enhanced photocatalytic activity of the 2D/1D NiO/CdS heterojunction can be attributed to the efficient separation of photogenerated charge carriers driven from the formation of p-n NiO/CdS heterojunction. This study paves a new way to develop 2D p-type NiO nanosheets-decorated n-type semiconductor photocatalysts for photocatalytic applications.

## Introduction

Photocatalysis has been recognized as an auspicious strategy to confront the energy and environmental crises (Shao et al., 2019; Wang et al., 2019). Developing low-cost and highly efficient heterojunction photocatalysts is pivotal for the large-scale commercialization of photocatalytic hydrogen generation. Since TiO_2_ was first reported as a promising semiconductor for water splitting (Fujishima and Honda, 1972), a significant number of semiconductor photocatalysts, such as metal sulfides (Chandrasekaran et al., 2019), metal oxides (Kumaravel et al., 2019), g-C_3_N_4_ (Rhimi et al., 2020), and metal-organic frameworks (Luo et al., 2020), have been explored for photocatalytic water splitting.

Cadmium sulfide (CdS), as a visible-light photocatalyst, has attracted tremendous attention due to its suitable bandgap and band-edge position for water splitting (Xu et al., 2018; Ruan et al., 2020). Nevertheless, the photoactivity of CdS was greatly limited owing to its terrible photostability and photo-corrosion and rapid recombination of charge carriers (Zhou X. et al., 2017; Kuang et al., 2020). To address these issues, some strategies have been applied to improve its photocatalytic activity, such as co-catalyst modifying (Liu W. et al., 2018), structure and morphology controlling (Vaquero et al., 2017; Jiang et al., 2020), and heterojunction construction (Xu et al., 2020; Yang et al., 2020; Ding et al., 2021). In particular, the fabrication of semiconductor–semiconductor heterojunctions has turned out to be an effective tactic to broaden the light-response range and accelerate the separation of electron-hole pairs (Liu J. et al., 2018; Zhai et al., 2018; Lu X. et al., 2020). Notably, the p-n junction has proven to be an efficient approach to boost the photoactivity on the account that the formed internal electric field at the interface can promote the separation of electron-hole pairs (Zhang et al., 2018; Tang et al., 2019). For instance, the p-n NiSe_2_/CdS composites displayed a 2.7-fold higher photocatalytic hydrogen production rate than pristine CdS due to their effective separation and transfer of charge carriers (Chen et al., 2019). Zhang et al. (2013) reported that the p-n NiS/CdS composites loading with 5 mol% NiS exhibited improved photocatalytic hydrogen generation activity. Furthermore, Wang L. et al. (2018) reported that the p-n Cu_2_O/CdS heterojunction photoelectrode display improved photoactivity compared to CdS because the formed p-n junction facilitates the separation and transfer of photoinduced charges. Therefore, incorporating a suitable and stable p-type semiconductor into CdS can ensure a p-n heterojunction for improved photocatalytic performance.

Nickel oxide (NiO), as a low-cost and earth-abundant p-type semiconductor, has attracted significant attention in the field of photocatalysis, including photocatalytic degradation (Ahmad et al., 2018; Sabzehmeidani et al., 2018), photocatalytic CO_2_ reduction (Chen et al., 2018), and photocatalytic hydrogen evolution (Shi et al., 2019; Lin et al., 2020). However, many previous studies mainly focused on the NiO nanoparticles; the 2D sheet-like NiO nanosheets for photocatalytic application were seldom discussed. The p-type NiO nanosheets with a large surface area can provide more active sites and inhibit the aggregation of the photogenerated carrier, which is favorable for the enhanced photocatalytic performance. Hence, it is highly desirable to explore 2D NiO nanosheet-based p-n heterojunction photocatalysts for hydrogen production.

In this study, the NiO/CdS composite composed of p-type 2D NiO nanosheets and the n-type 1D CdS nanorods were prepared using a solution-phase hybridization approach. The 5% NiO/CdS composite exhibited a prominently enhanced photocatalytic hydrogen evolution activity when compared with CdS nanorods. In addition, the photocatalytic mechanism of the 2D/1D NiO/CdS p-n heterojunction was proposed based on the photoluminescence (PL) and photoelectrochemical (PEC) tests. This current research highlights the advantages of the 2D/1D p-n heterojunctions toward improved photocatalytic hydrogen generation performance.

## Experimental Section

### Preparation

#### Preparation of CdS Nanorods

First, 2 mmol of cadmium acetate [Cd(OAc)_2_·2H_2_O] and 4 mmol of thiourea (CH_4_N_2_S) were initially dissolved into 50 ml of ethylenediamine. The mixture was then placed in a 100-ml Teflon-lined autoclave and heated to 180°C for 16 h. The product was obtained by using centrifugation and washed with DI water and ethanol before vacuum drying.

#### Preparation of NiO Nanosheets

The NiO nanosheets were prepared by the calcination of α-Ni(OH)_2_ nanosheets. Firstly, α-Ni(OH)_2_ nanosheets were synthesized through a modified method based on a previous report (Wang D. et al., 2018). Typically, 1 mmol of nickel nitrate hexahydrate [Ni(NO_3_)_2_·6H_2_O] and 2 mmol of urea (CH_4_N_2_S) were dissolved in 35 ml of ethanol. The mixture was then transferred into 50 ml of Teflon-lined autoclave and kept at 120°C for 8 h. The α-Ni(OH)_2_ nanosheets were obtained after centrifugation, washed, and vacuumed dried. Finally, the NiO nanosheets were prepared by annealing the as-obtained α-Ni(OH)_2_ nanosheets at 350°C for 1 h under air with a heating rate of 1°C/min.

#### Preparation of NiO/CdS Composites

The NiO/CdS composites were synthesized through a solution-phase method. Firstly, NiO nanosheets and CdS nanorods were dispersed in 40 ml of ethanol. Then, the mixture was magnetically stirred for 12 h. Finally, the product was obtained after the mixture was washed and vacuum dried. The final products were denoted as *x*% NiO/CdS, in which *x*% represented the mass ratio of NiO to the NiO/CdS composite.

## Characterization

The crystal phases of the samples were carried out using a powder X-ray diffractometer (XRD, Smartlab-3KW). The scanning electron microscopy (SEM) image was obtained using a HITACHI S-3400 N microscope. The images from transmission electron microscopy (TEM) were acquired from a TECNAI F-30 microscope (FEI Company, Hillsboro, OR, United States). The UV-visible spectra of the samples were obtained on a PerkinElmer, Lambda 750. The photoluminescence (PL) spectra were obtained using FL3C-111 (HORIBA Instruments Inc.). The x-ray photoelectron spectroscopy (XPS) measurements were acquired using an ESCALAB 250Xi spectrometer. For the photoelectrochemical (PEC) tests, photocurrent responses, electrochemical impedance spectra (EIS), and Mott–Schottky plots were performed on a Bio-Logic VSP-300 potentiostat in a 0.1 M Na_2_SO_4_ solution, where a Pt sheet, Ag/AgCl, and photocatalyst-coated ITO glass were used as the counter electrode, reference electrode, and working electrode, respectively.

## Photocatalytic Measurements

The photocatalytic H_2_ production activities were measured on a closed-circulation apparatus. Typically, the photocatalyst (40 mg) was suspended into a 0.2 M Na_2_S/0.35M Na_2_SO_3_ aqueous solution (60 ml). The mixture was then irradiated under a 300 W Xe lamp (CEL-HXF300, Beijing Aulight, λ > 420 nm). The amount of H_2_ was determined using gas chromatography (GC-7920) using a thermal conductivity detector. After the H_2_ in the previous cycle was fully removed, the cycling tests for the photocatalytic H_2_ production were carried out under identical conditions.

## Results and Discussion

### Synthesis and Characterization

**Figure 1:**
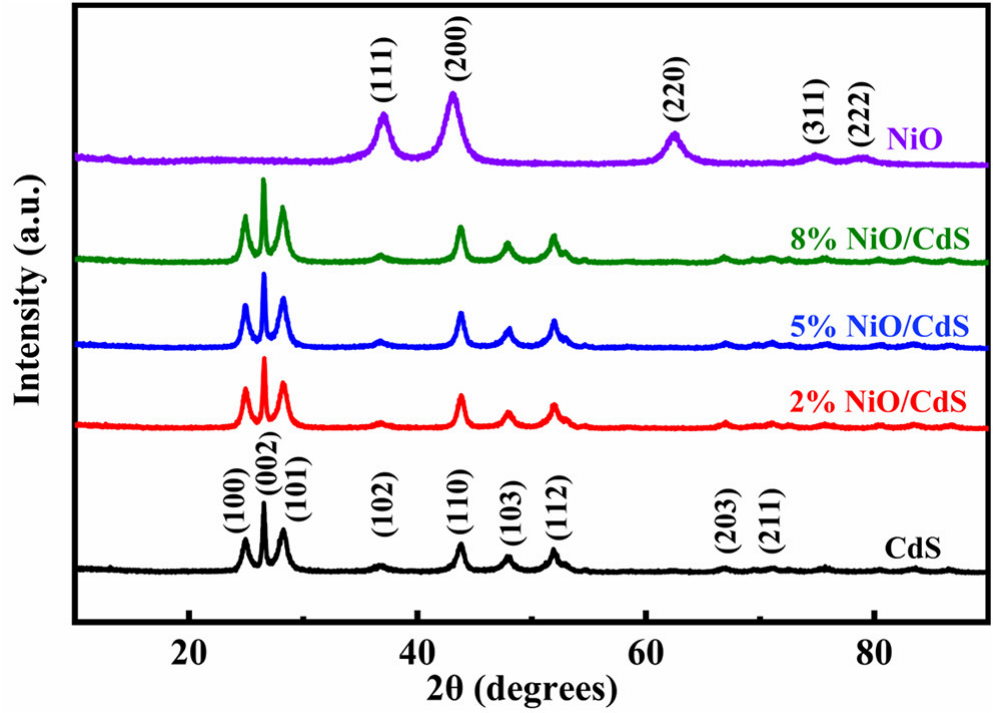
XRD patterns of pristine NiO, cadmium sulfide (CdS), NiO/CdS composites with different contents of NiO nanosheets.

Figure 1 shows the XRD patterns of pure CdS, NiO, and NiO/CdS composites with different NiO contents. The diffraction peaks of the as-obtained CdS sample can be well-indexed to those of hexagonal CdS (JCPDS No. 77-2306) (Zhang S. et al., 2012; Shen et al., 2020). All of the diffraction peaks of the NiO sample matched with cubic NiO (JCPDS No. 89-7130), indicating the successful preparation of NiO by the calcination of α-Ni(OH)_2_ (Supplementary Figure 1, JCPDS No. 22-0444). Moreover, the NiO/CdS photocatalysts exhibit similar diffraction peaks to CdS, which can be attributed to the low content of NiO (Chen et al., 2019; Tang et al., 2019). To reveal the coexistence of NiO and CdS in the composite, the following SEM, TEM, and XPS measurements were performed.

**Figure 2:**
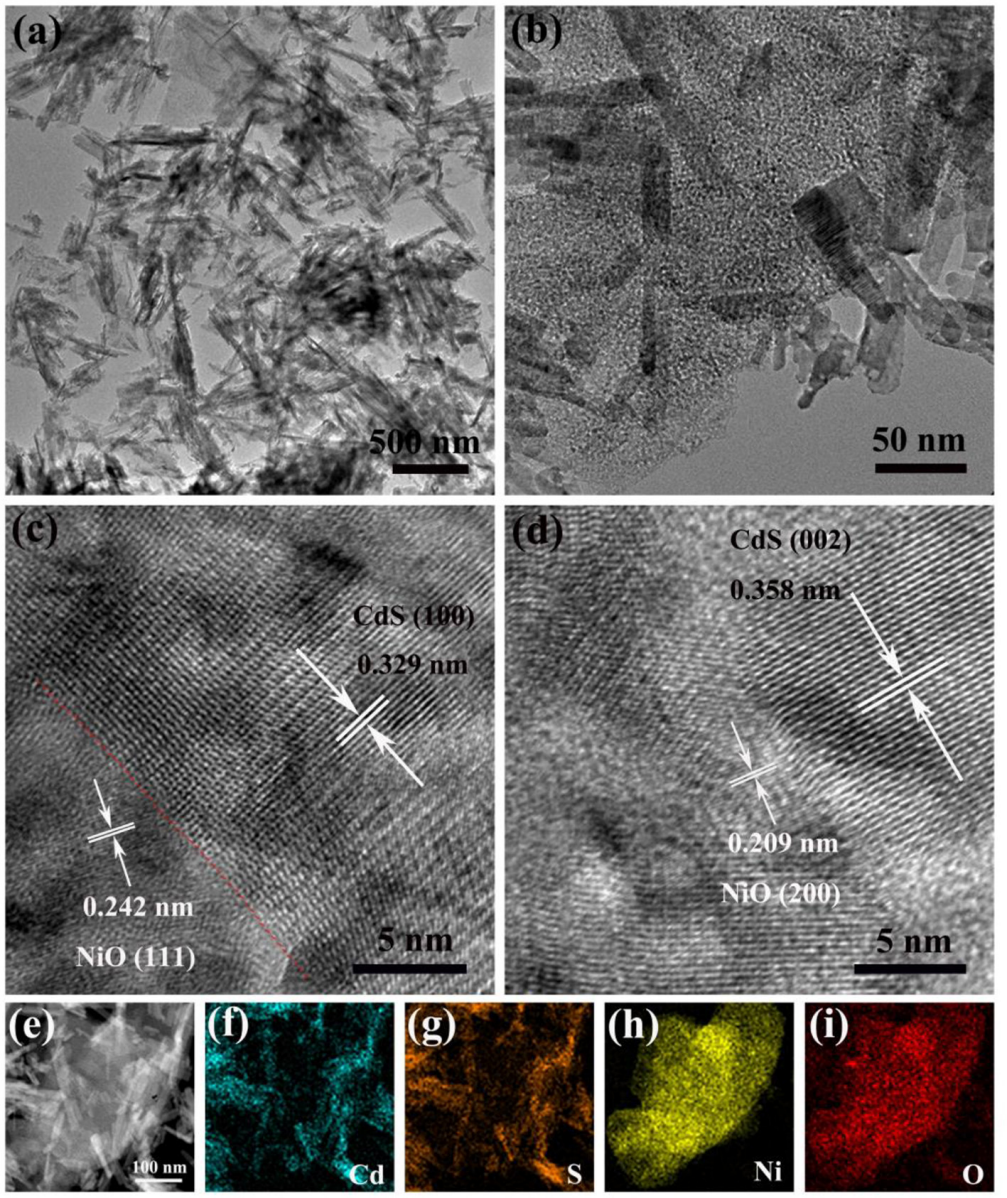
**(a,b)** TEM and **(c,d)** HRTEM images of 5% NiO/CdS; **(e)** HAADF and **(f–i)** the corresponding elemental mapping images of Cd, S, Ni, and O of 5% NiO/CdS.

The morphology and structure of photocatalysts were identified by SEM. As shown in Supplementary Figures 2, 3, the NiO could retain the 2D sheet-like morphologies of the α-Ni(OH)_2_ after the calcination process. In the meantime, the CdS exhibited rod-like morphology with a length of 100–500 nm (Supplementary Figure 4). It can be observed in the SEM image of 5% NiO/CdS (Supplementary Figure 5) that the CdS nanorods were uniformly deposited onto the NiO nanosheets after hybridization treatment. To further confirm the microstructures and interfaces of the NiO/CdS composite, TEM and high-resolution TEM (HRTEM) were carried out. As shown in Figures 2a,b, the NiO nanosheets were intimately attached to the CdS nanorods. Moreover, the continuous lattice fringes (Figure 2c) in the HRTEM reveal that the lattice fringe spacings of 0.329 and 0.242 nm were assigned to (100) plane of the CdS nanorods and (111) plane of the NiO nanosheets, respectively. Similarly, CdS (002) and NiO (200) also can be observed in the HRTEM image (Figure 2d), indicating the presence of an intimate interface between the NiO nanosheets and the CdS nanorods. Such tight contact in the NiO/CdS composite would favor the separation of photogenerated charge carriers (Chen et al., 2019; Lu Y. et al., 2020; Zhao et al., 2020). Furthermore, the high-angle annular dark-field (HAADF) and the corresponding scanning TEM energy dispersive x-ray (STEM-EDX) elemental mapping images of NiO/CdS (Figures 2e–i) illustrated that Ni and O were of homogeneous distribution in the NiO nanosheets, while Cd and S were in the rod-like CdS structures, implying the coexistence of 2D NiO nanosheets and 1D CdS nanorods in the NiO/CdS composite.

**Figure 3:**
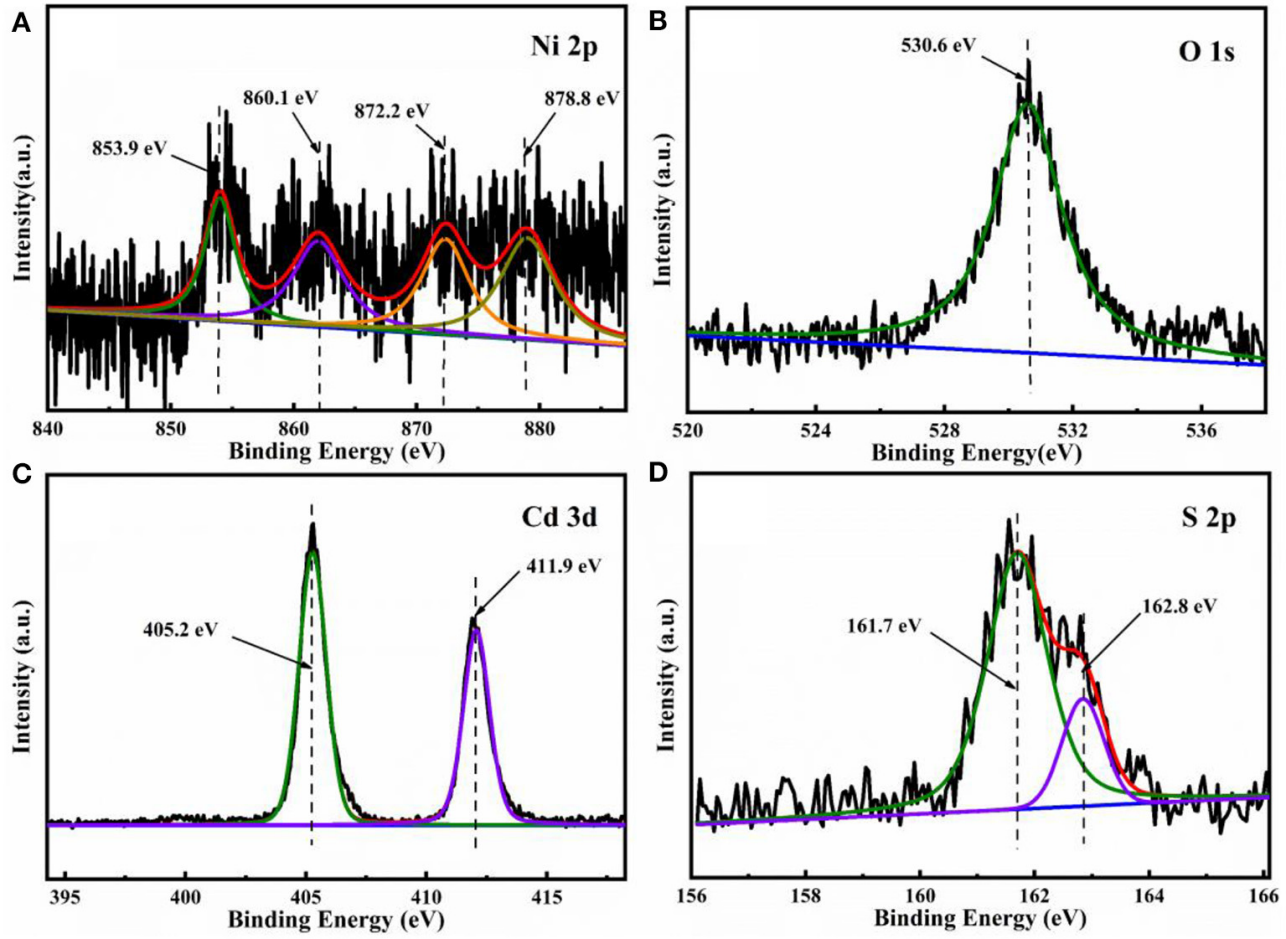
XPS spectra of **(A)** Ni 2p, **(B)** O 1s, **(C)** Cd 3d, and **(D)** S 2p of the 5% NiO/CdS sample.

The XPS further analyzed the elemental composition and chemical states of the 5% NiO/CdS sample. Two peaks appear at 853.9 and 872.2 eV in the Ni 2p spectrum (Figure 3A), corresponding to the binding energies of Ni^2+^ 2p_3/2_, and 2p_1/2_; the peaks at 860.1 and 878.8 eV could be attributed to the satellite peaks (Yang et al., 2019; Wu et al., 2020). As shown in Figure 3B, the peak located at 530.6 eV could be attributed to the lattice oxygen of NiO (Wang et al., 2010; Liu et al., 2012; Hu et al., 2018). Figure 3C displays the high-resolution XPS spectra of Cd 3d; the binding energies at 405.2 and 411.9 eV correspond to the Cd 3d_5/2_ and Cd 3d_3/2_ of the Cd^2+^ in CdS nanorods, respectively (Bai et al., 2010; Zhou P. et al., 2017; Behera et al., 2019). The XPS spectrum of S (Figure 3D) presents two peaks at 162.8 and 161.7 eV that were assigned to the characteristic binding energies of S 2p_1/2_ and S 2p_3/2_ of S^2−^. The XPS results further indicate the construction of the 2D/1D NiO/CdS composite, which were well-matched with the SEM (Supplementary Figure 5) and TEM images (Figure 2).

**Figure 4:**
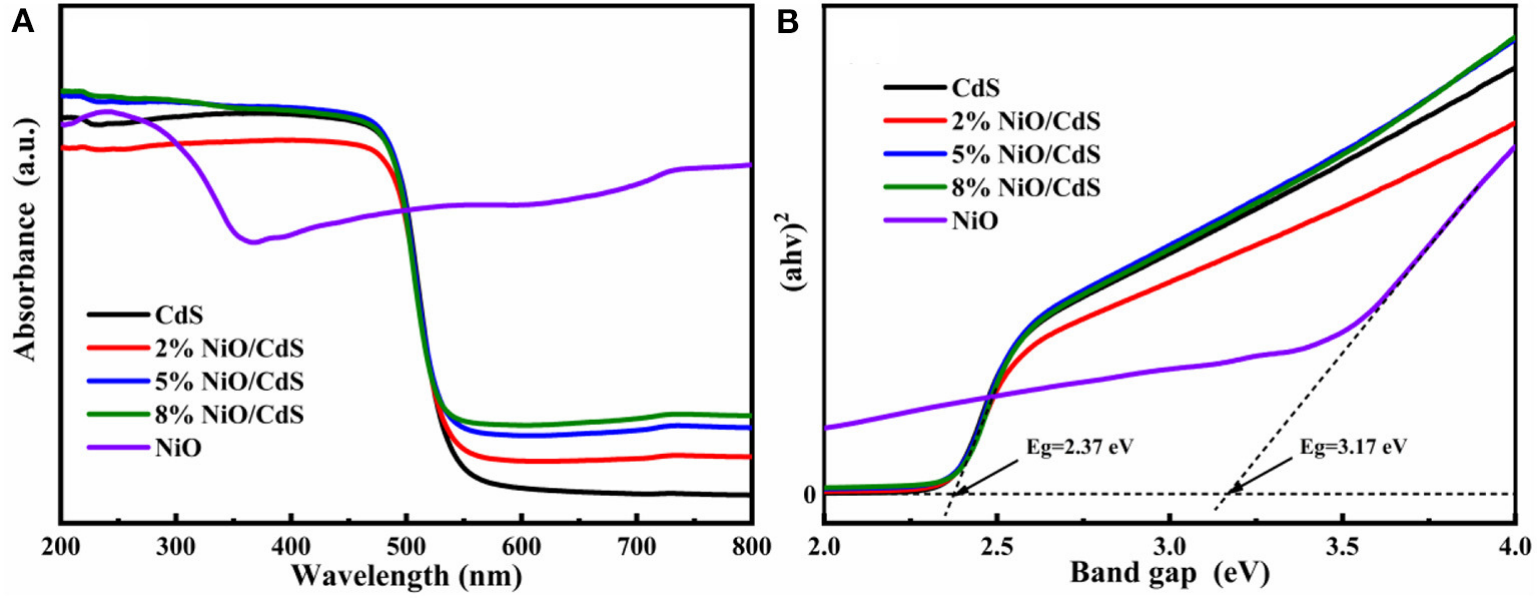
**(A)** UV-vis DRS and **(B)** Tauc plots of the CdS, NiO, and NiO/CdS composites.

Figure 4A displays the UV-vis diffuse reflectance spectra (DRS) of the CdS, NiO, and NiO/CdS composites. The NiO/CdS composites present a similar absorption edge to pristine CdS, indicating that the intrinsic bandgap of CdS could be retained after loading a small amount of NiO content (Wang L. et al., 2018; Chen et al., 2019). Besides, a significantly increased absorption in the range of 550–800 nm for the NiO/CdS composites with the increasing NiO content could be assigned to the strong absorption of the NiO nanosheets. The bandgap energies of CdS and NiO were measured based on the transformed Kubelka–Munk function (Carbone et al., 2017; Chen et al., 2019; Xu et al., 2021). According to the Tauc plots (Figure 4B), the bandgap of the CdS nanorods and NiO nanosheets were calculated to be 2.37 and 3.17 eV, respectively (Kandi et al., 2020).

**Figure 5:**
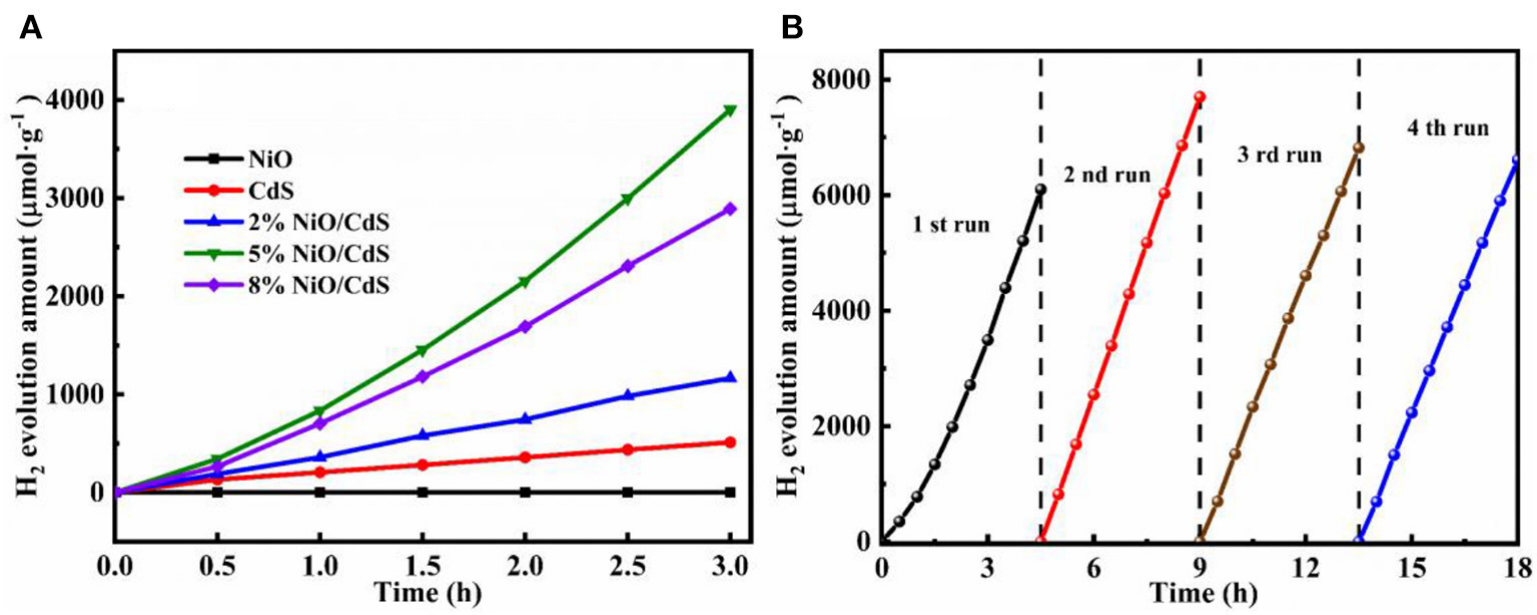
**(A)** Photocatalytic H_2_ production activities of CdS, NiO, and NiO/CdS composites. **(B)** Recycling tests of photocatalytic H_2_ generation over 5% NiO/CdS.

The photocatalytic hydrogen generation performance of the as-prepared photocatalysts was measured under visible light. As illustrated in Figure 5A, the NiO nanosheets exhibit trace amounts of hydrogen evolution, which could be attributed to the fast recombination of the photogenerated charge carriers. The pristine CdS displays a hydrogen production rate of 150 μmol h^−1^ g^−1^. Notably, the hydrogen generation amount of all of the NiO/CdS photocatalysts was higher than that of CdS. It is impressive that the optimal 5% NiO/CdS sample exhibited a hydrogen generation rate of 1,300 μmol h^−1^ g^−1^, which is more than eight times higher than that of pristine CdS. By further increasing the NiO loading up to 8 wt.%, the hydrogen evolution activity was slightly decreased due to the shielding effect caused by the excessive amount of NiO (Zeng et al., 2019). Moreover, as displayed in Supplementary Figure 6, the 5% NiO/CdS samples using UV-vis light source (λ > 350 nm) exhibited enhanced activity when compared to CdS and NiO, further implying that the formed 2D/1D NiO/CdS heterojunction was beneficial for the separation and transfer of photoexcited electron-hole pairs. Meanwhile, to further evaluate the recycling stability of the 5% NiO/CdS composites, four cycles of photocatalytic hydrogen production were carried out. As depicted in Figure 5B, the NiO/CdS composite photocatalysts exhibit superior stable hydrogen generation activity during the multi-cycle photocatalytic reaction within 18 h. Furthermore, the crystal structure of the used 5% NiO/CdS sample was studied. As revealed by the XRD patterns (Supplementary Figure 7), the crystal phase basically remained unchanged after the cycling tests. Such results demonstrate the excellent photoactivity and stability of the NiO/CdS composite.

**Figure 6:**
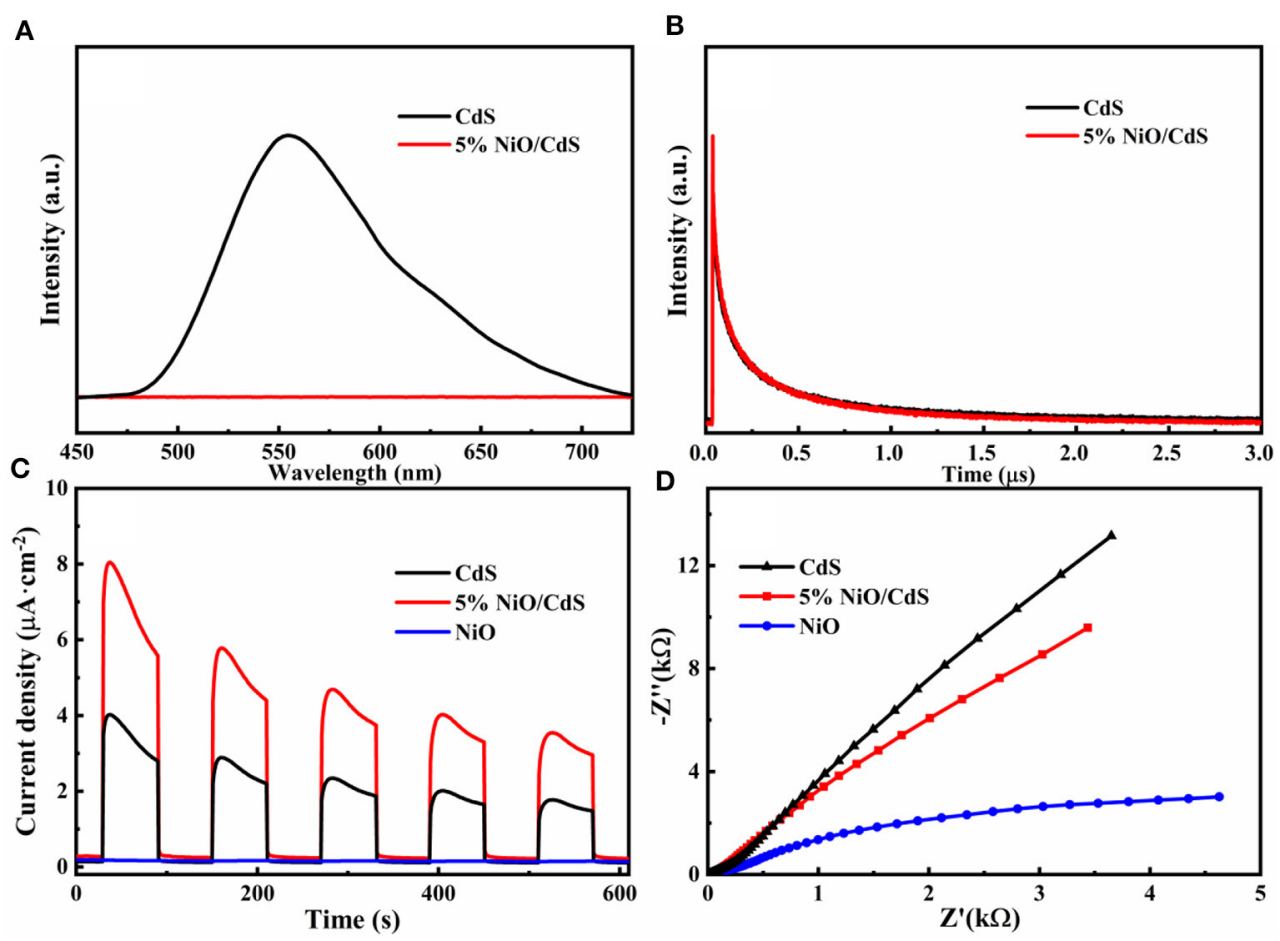
**(A)** PL and **(B)** TRPL spectra of CdS and NiO/CdS. **(C)** Photocurrent response curves and **(D)** EIS Nyquist plots of pristine CdS, NiO, and 5% NiO/CdS samples.

To deeply investigate the effect of NiO nanosheets on the enhanced photocatalytic performance of CdS, the PL and the PEC tests of the as-obtained photocatalysts were carried out. As illustrated in Figure 6A, pristine CdS shows a pronounced emission peak, revealing its rapid charge carrier recombination. Notably, the PL intensity of the 5% NiO/CdS photocatalysts was significantly decreased, implying that the enhanced separation efficiency of photogenerated carriers was achieved after loading the NiO nanosheets (Kandi et al., 2017; Li et al., 2020). Furthermore, the time resolved PL (TRPL) spectra were conducted to unveil the dynamic transfer of charge carriers of the NiO/CdS composite. As illustrated in Figure 6B and Supplementary Table 1, the PL lifetime of the 5% NiO/CdS composite (1.93 μs) is higher than that of the pristine CdS (1.73 μs), illustrating the effective charge transfer in the NiO/CdS nanohybrids (Hao et al., 2019; Kandi et al., 2019; Peng et al., 2021; Yang et al., 2021). For PEC measurements, as displayed in Figure 6C, the photocurrent response of the 5% NiO/CdS composite is significantly higher than that of the pristine CdS, indicating that the 2D NiO nanosheets can effectively promote the charge separation in the NiO/CdS photocatalyst (Chai et al., 2018; Shao et al., 2019; Chen et al., 2020). Also, as revealed by the EIS Nyquist plots (Figure 6D), the radius of the 5% NiO/CdS composites is significantly smaller than that of the CdS, suggesting that the charge transfer resistance was decreased considerably after incorporating NiO nanosheets, which was beneficial in improving the photocatalytic activity (Zhang J. et al., 2012; Kuang et al., 2020).

**Figure 7:**
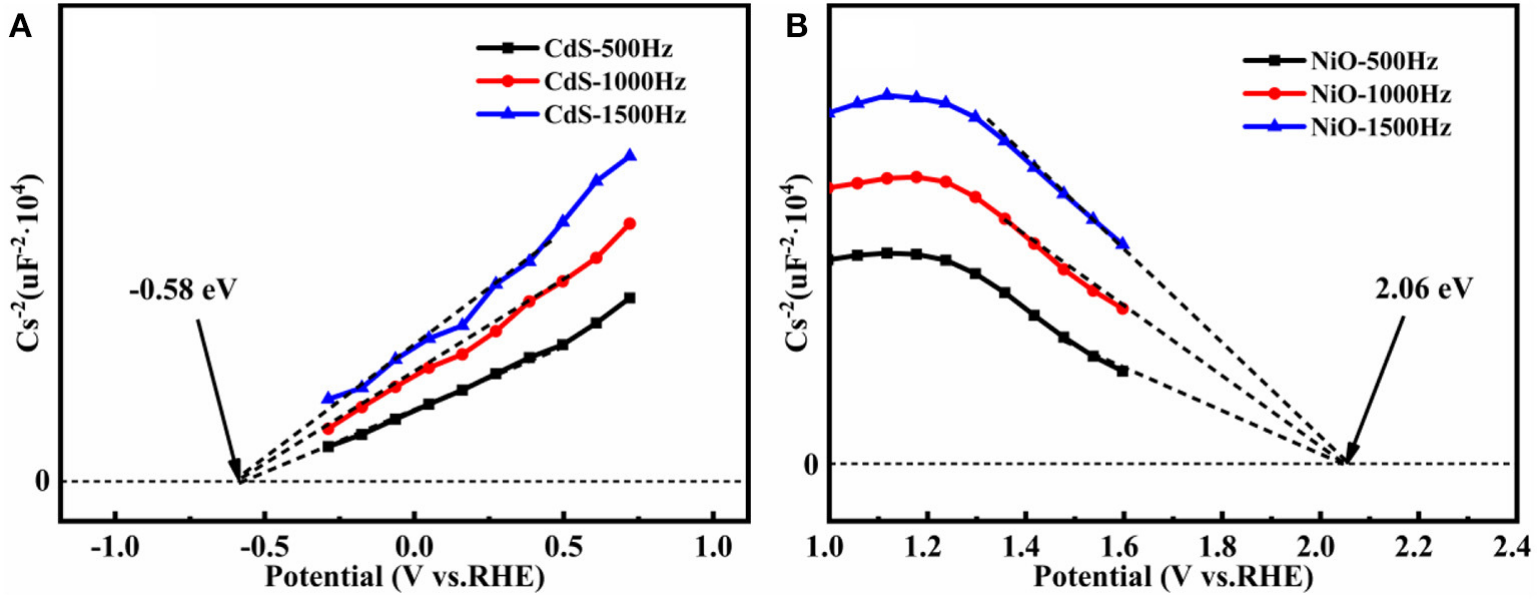
Mott–Schottky plots of **(A)** CdS and **(B)** NiO.

As depicted in Figure 7, the Mott–Schottky curves of CdS and NiO at 500, 1,000, and 1,500 Hz were measured, according to the equation (E_RHE_ = E_Ag/AgCl_ + 0.059 × pH + 0.205) for the conversion of Ag/AgCl to the reversible hydrogen electrode (RHE) scale. The positive and negative slopes of CdS and NiO were observed, corresponding to n-type and p-type semiconductors, respectively. Furthermore, based on the Mott–Schottky plots, the conduction band (CB) of CdS and the valence band (VB) of NiO were estimated to be ~−0.58 and 2.06 eV, respectively (Qiu et al., 2017; Chen et al., 2019; Ruan et al., 2020). Combined with the bandgap values of CdS and NiO derived from Tauc plots (Figure 4B), the VB and the CB of CdS were located to be at 1.79 and −1.11 eV, respectively.

**Figure 8:**
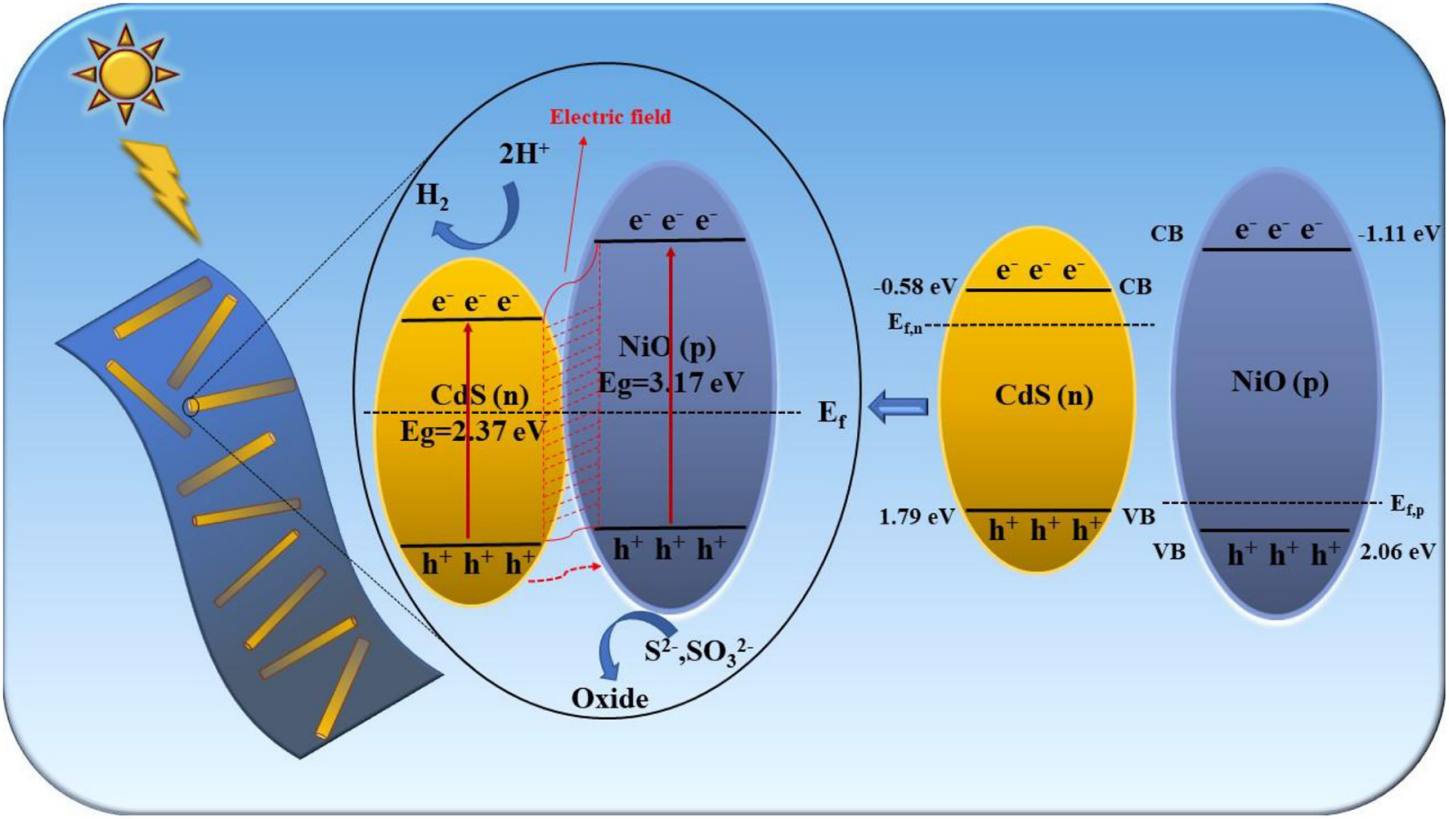
The possible photocatalytic mechanism for NiO/CdS system.

A possible photocatalytic hydrogen evolution mechanism of the 2D/1D NiO/CdS heterojunction is proposed in Figure 8. After coupling the n-type CdS nanorods with the p-type NiO nanosheets, the charge carriers at the interphase boundary undergo redistribution to balance the Fermi level (Chen et al., 2011, 2019). Thus, the energy band (VB and CB) of p-type NiO was raised to a location higher than that of CdS, and the formed p-n junction was favorable to build the inner electric field at the interface between CdS and NiO (Wang L. et al., 2018; Shi et al., 2019). When the p-n NiO/CdS is irradiated under visible light, the electrons were excited from the VB to their CB in CdS and NiO. The photoexcited electrons in the CB of NiO could migrate to that of CdS, whereas the holes in the CdS were transferred to the VB of NiO. Therefore, the construction of the p-n 2D/1D NiO/CdS heterojunction could efficiently promote the separation and migration of the photoexcited electron-hole pairs (Xiao et al., 2020; Zhao et al., 2020), resulting in improved photocatalytic hydrogen production activity.

## Conclusions

In summary, the CdS nanorods were uniformly anchored onto the NiO nanosheets through a solution-phase hybridization method. The 2D/1D NiO/CdS heterojunction photocatalyst unveiled efficient photoactivity toward hydrogen production under visible light. The resultant 5% NiO/CdS exhibited a photocatalytic hydrogen production rate of 1,300 μmol h^−1^ g^−1^, which is more than eight times higher than that of the CdS nanorods. Moreover, the NiO/CdS composite displays excellent stability over four cycles of photocatalytic hydrogen production. According to the results from the PL, the TRPL, the PEC, Mott–Schottky plots, the improved photocatalytic hydrogen evolution performance was mainly attributed to the efficient separation of charge carriers caused by the formed p-n NiO/CdS heterojunction. This study provides new opportunities in improving photocatalytic activity by constructing 2D/1D p-n heterojunction photocatalysts.

## Data Availability Statement

The raw data supporting the conclusions of this article will be made available by the authors, without undue reservation.

## Author Contributions

LW and DZ planned the experimental work, wrote the manuscript, and helped in the analysis. ZX, QZ, and HZ methodology, formal analysis, and data curation. DZ, TF, and YW review, editing, and funding acquisition. All authors contributed to the article and approved the submitted version.

## Conflict of Interest

The authors declare that the research was conducted in the absence of any commercial or financial relationships that could be construed as a potential conflict of interest.
