# Supplementary material for: NiO Nanosheets Coupled With CdS Nanorods as 2D/1D Heterojunction for Improved Photocatalytic Hydrogen Evolution
Source: Front Chem. 2021 Apr 15;9:655583. doi: 10.3389/fchem.2021.655583 (PMC8082420; doi:10.3389/fchem.2021.655583)
Supplement: Supplementary file 1 [file Data_Sheet_1.PDF]

Supporting Information

NiO nanosheets coupled with CdS nanorodsas 2D/1D heterojunction for improved photocatalytic hydrogen evolution

Lin Wei[1], DeqianZeng[1]*, Zongzhuo Xie[1], Qingru Zeng[1], Hongfei Zheng[2], Toyohisa Fujita[1]*and Yuezhou Wei[1]

[1]Guangxi Key Laboratory of Processing for Non-ferrous Metals and Featured Materials, School of Resources, Environment and Materials, Guangxi University, Nanning 530004, China.

[2]Collaborative Innovation Center of Chemistry for Energy Materials, College of Materials, Xiamen University, Xiamen 361005, Fujian, China.

[*]Corresponding author.

Email: dqzeng@gxu.edu.cn; fujitatoyohisa@gxu.edu.cn

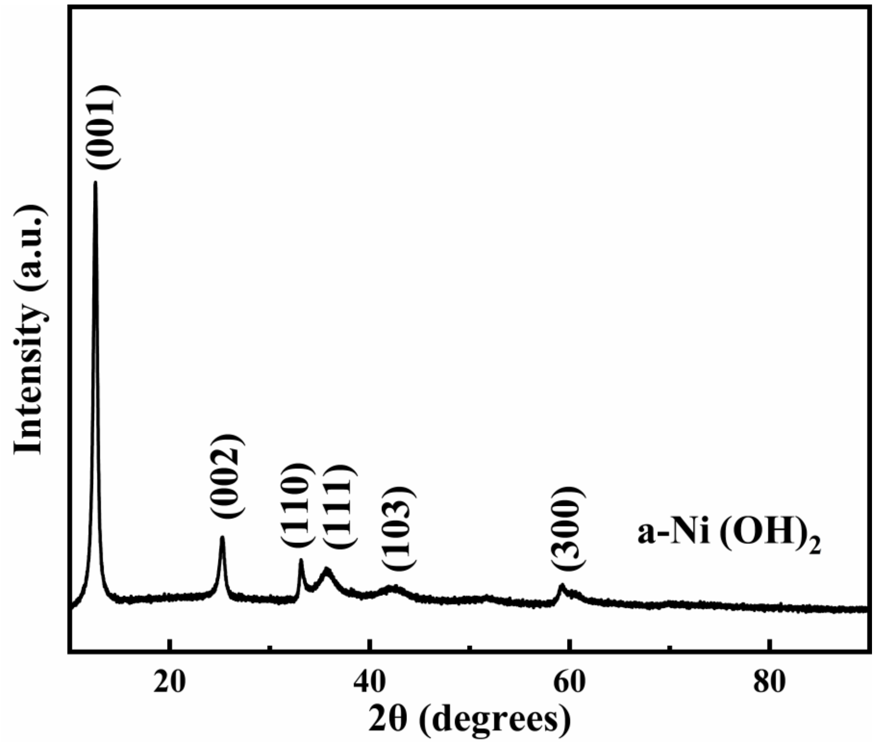

Fig. S1. XRD pattern of α-$Ni(OH)_2$ nanosheets.

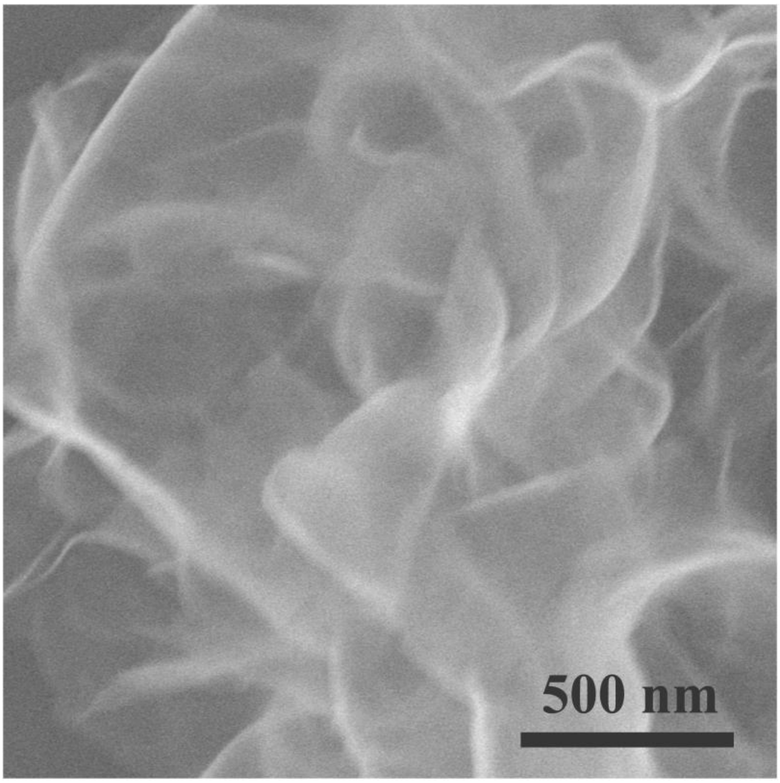

Fig. S2. SEM image of α-$Ni(OH)_2$ nanosheets.

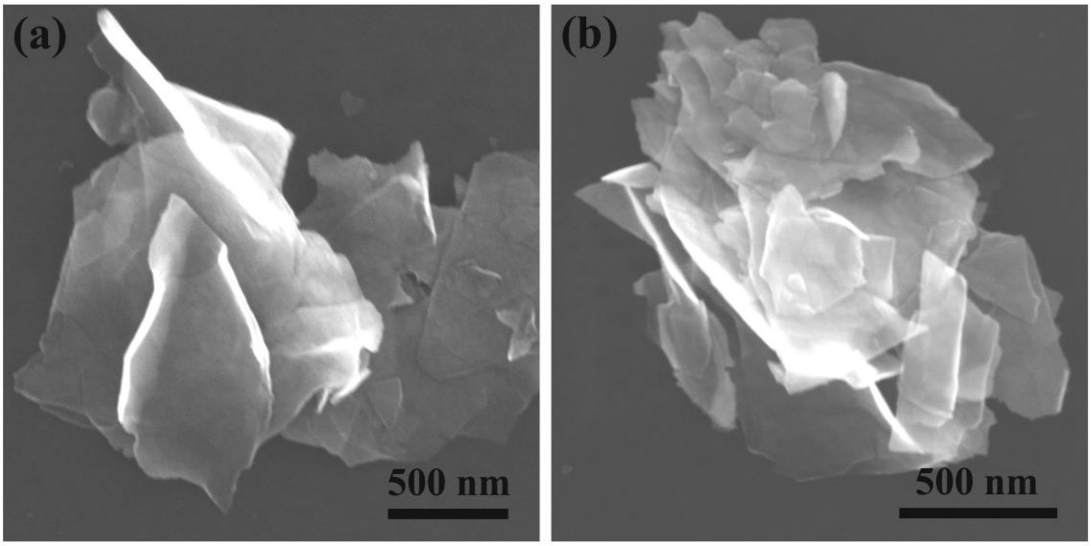

Fig. S3. SEM image of NiO nanosheets.

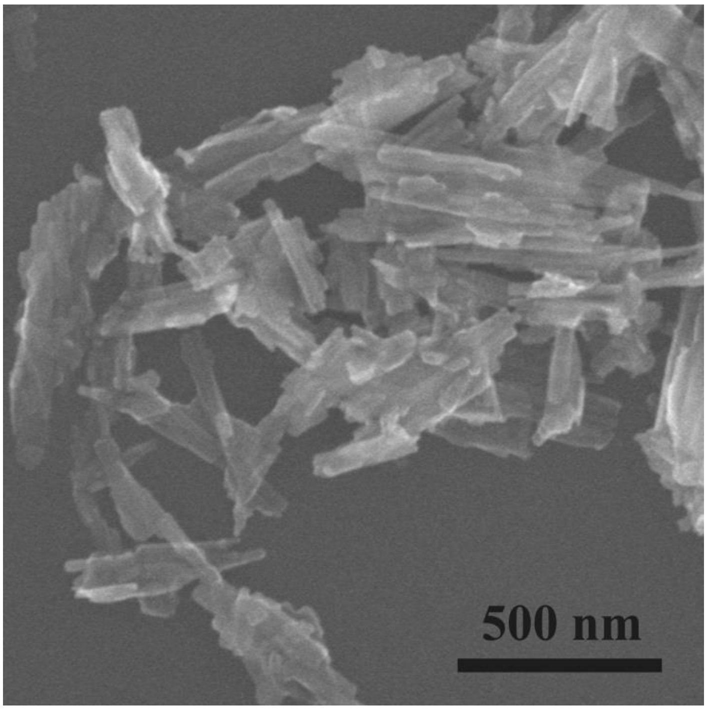

Fig. S4. SEM image of CdS nanorods.

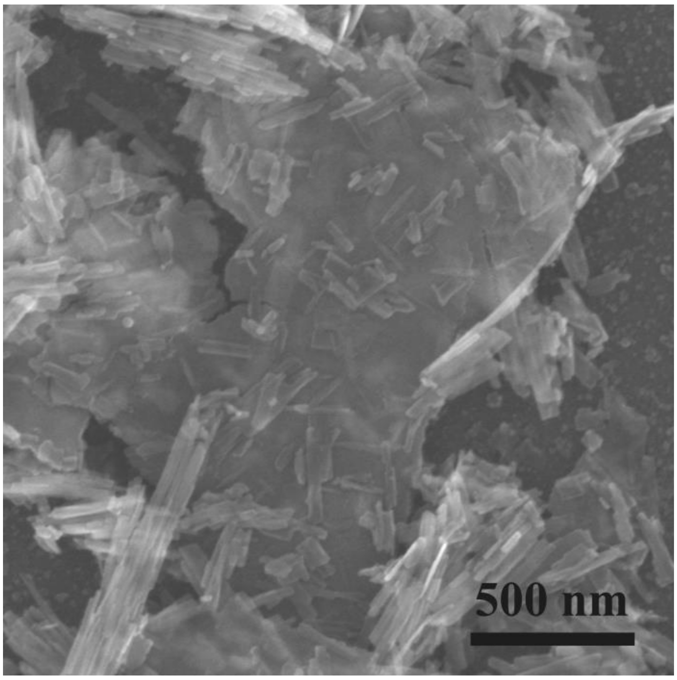

Fig. S5. SEM image of 5% NiO/CdS sample.

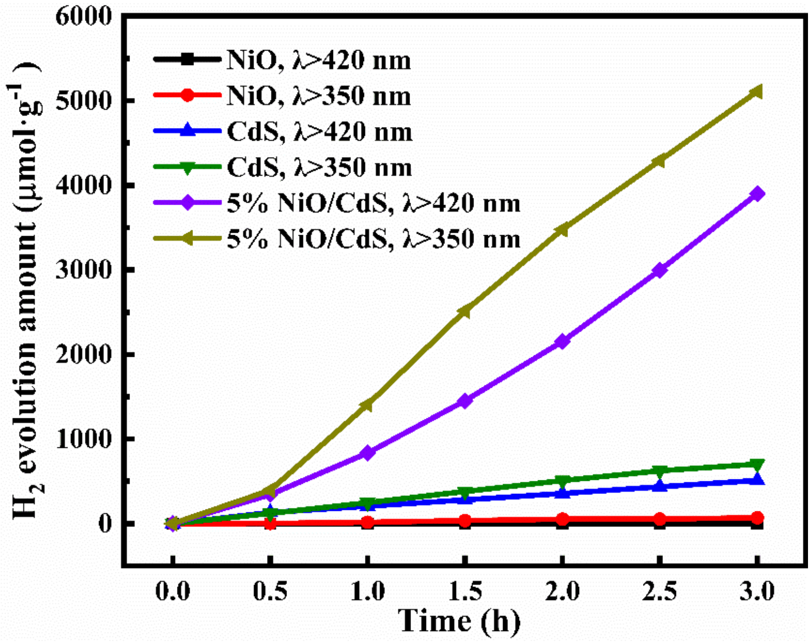

Fig. S6. Photocatalytic H_2 production activities of CdS, NiO, and 5% NiO/CdS using UV-Vis and visible light source irradiation.

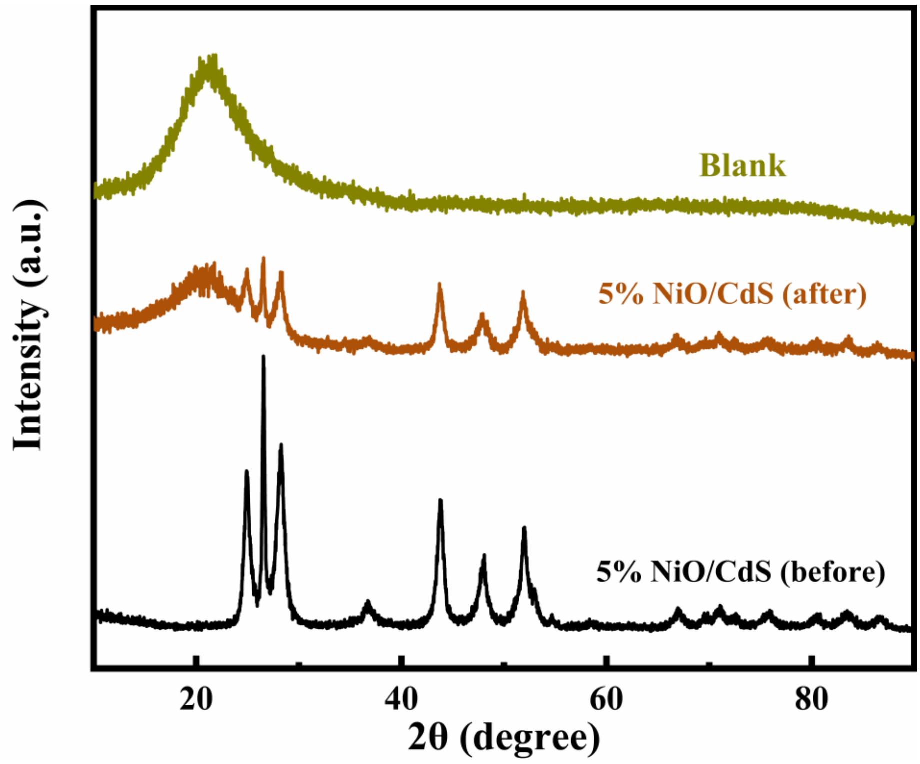

Fig. S7. XRD patterns of 5% NiO/CdS sample before and after cycling tests along with the blank quartz holder.

Table S1 Decay parameters of pristine CdS and 5 %NiO/CdS composite.

**Samples**	**Lifetime, T (ns)**	**Rel (%)**	**T_{ave} (ns)**
**CdS**	T1=111.8<br>T2=562.6<br>T3=14.5	B1=24.95<br>B2=71.77B3=3.28	173.4
**5 %NiO/CdS**	T1=137.9 T2=651.2<br>T3=21.9	B1=27.34<br>B2=67.98<br>B3=4.68	193.6
